# Immune-modulating Activity of Hydrogel Microparticles Contributes to the Host Defense in a Murine Model of Cutaneous Anthrax

**DOI:** 10.3389/fmolb.2017.00062

**Published:** 2017-08-28

**Authors:** Allison L. Teunis, Taissia G. Popova, Virginia Espina, Lance A. Liotta, Serguei G. Popov

**Affiliations:** ^1^Department of Molecular Microbiology, National Center for Biodefense and Infectious Diseases, School of Systems Biology, George Mason University Manassas, VA, United States; ^2^Department of Molecular Microbiology, Center for Applied Proteomics and Molecular Medicine, School of Systems Biology, George Mason University Manassas, VA, United States

**Keywords:** anthrax, microparticles, hydrogel, chemokines, mice, survival

## Abstract

We recently reported that the open-mesh (0.7 μ) polyacrylamide microparticles (MPs) with internally-coupled Cibacron affinity dye demonstrate protective effect in mice challenged into footpads with high doses (200 LD50) of anthrax (Sterne) spores. A single injection of MPs before spore challenge reduces inflammatory response, delays onset of mortality and promotes survival. In this study, we show that the effect of MPs was substantially increased at the lower spore dose (7 LD50). The inflammation of footpads was reduced to the background level, and 60% of animals survived for 16 days while all untreated infected animals died within 6 days with strong inflammation. The effects of MPs were promoted when the MPs were loaded with a combination of neutrophil-attracting chemokines IL-8 and MIP-1α which delayed the onset of mortality in comparison with untreated mice for additional 8 days. The MPs were not inherently cytotoxic against the bacteria or cultured murine Raw 264.7 cells, but stimulated these cells to release G-CSF, MCP-1, MIP-1α, and TNF-α. Consistent with this finding the injection of MPs induced neutrophil influx into footpads, stimulated production of TNF-α associated with migration of pERK1/2-positive cells with the Langerhans phenotype from epidermis to regional lymph nodes. Our data support the mechanism of protection in which the immune defense induced by MPs along with the exogenous chemokines counterbalances the suppressive effect caused by anthrax infection.

## Introduction

Engineered nano- and micro-particles (MPs) find a fast-growing variety of applications in industry, biology, and medicine. For instance, the MPs can be used as active antimicrobial agents and drug delivery vehicles for antibacterial therapeutics. Some types of MPs display their effects through direct interaction with the pathogen while others are capable of inducing protection of the host against infection indirectly through stimulation or suppression of the immune cell responses (Look et al., 2010; Qasim et al., 2014; Gupta et al., 2016). The interaction of MPs with the immune system can either cause a desired effect or promote immunopathology raising concerns about their safe use. MPs are capable of targeting different cells types present in tissues contacting with the external environment such as, the skin, inner lining of the nose, lungs, stomach, and intestines. One of the most likely biologically relevant MP targets are dendritic cells (DCs) due to a number of key DC properties, such as, their capacity to internalize foreign particles, transport them to lymph nodes, and present the associated antigens to other immune cells. A number of DC subpopulations (plasmacytoid, conventional, inflammatory) can be identified based on surface antigen phenotype and developmental origin. The skin contains a specialized epidermal DC type called the Langerhans cells (LCs) as well as distinct populations of dermal DCs (Tőke et al., 2014). The capacity of MPs to induce immune response or enhance presentation of MP-loaded antigens made them popular candidates as vaccine adjuvants. Adjuvants induce recruitment of various immune cells to the site of injection, some of which then traffic the antigen to the draining lymph nodes to induce specific immune responses (Mosca et al., 2008; McKee et al., 2009).

The mechanisms of immune activation by MPs are diverse and largely determined by their surface chemistry (Gupta et al., 2016). Many MPs are capable of inducing inflammatory pathways in DCs mediated by signaling *via* the extracellular signal-regulated kinase 1/2 (ERK1/2) which can be activated by growth factors, cytokines, stress factors, viral infections, carcinogens, and bacterial components such as, lipopolysaccharide (LPS; Karlson et al., 2013). pERK1/2 signaling in DCs has been shown to be involved in DC differentiation, survival, as well as regulation of inflammation (Rescigno et al., 1998; Verhaeghe et al., 2007; Arce et al., 2011). Several chemokine genes, including MIP-1α/CCL3 (macrophage inflammatory protein-1α), MCP-1/CCL2 (monocyte chemotactic protein-1), and CCL4/MIP-1β (macrophage inflammatory protein-1β), are up-regulated through activation of ERK pathway in DCs (Yan et al., 2007). Experiments with a diverse range of mouse models provide *in vivo* evidence indicating that conventional DCs of myeloid origin play an important role in the regulation of neutrophil homeostasis. Taking into account key functions of the neutrophil innate immune responses against many infectious diseases, the MPs have a potential to regulate protection of the host during infectious process through their effects on DCs (Charmoy et al., 2010). Nevertheless, only a handful of studies demonstrating immune modulation by MPs as therapeutic agents for infectious disease treatment has been reported (Seil and Webster, 2012; Qasim et al., 2014).

Recently, we employed polyacrylamide hydrogel MPs covalently coupled with Cibacron Blue (CB) affinity dye (later referred to as CK-MPs or MPs) to protect mice against infection with *Bacillus anthracis* (B.a.), an etiological agent of anthrax (Popova et al., 2016). In this model, our MPs accumulate in the regional draining lymph nodes (LNs) where they maintain biologically significant levels of immune-modulating chemokine (CK) release for more than 20 h (Popova et al., 2015). Mice represent a convenient animal model of anthrax because they are sensitive to the widely used attenuated Sterne strain 34F2. This strain retains the toxic mechanisms of the virulent strains but lacks a protective capsule which makes it more susceptible to phagocytes. The latter engulf B.a. spores and bring them to the draining LNs thus establishing the productive infectious process which, among other factors, is associated with the abnormally reduced CK signaling in the infected host (Paccani et al., 2007; van Sorge et al., 2008; Guichard et al., 2012). We hypothesized that this pathogenic impact of infection could be overcome using our MPs as vehicles for transport of inflammatory chemokines to LNs, with the purpose of enhancing migration of neutrophils and other immune cells to the site of infection. We found that the pretreatment of anthrax spore-challenged mice with chemokine-loaded MPs (CK-MPs) improved bacterial clearance and survival (Popova et al., 2016). Similar effects, albeit of lower degree, were found in the case of MPs without external CKs, raising a hypothesis that the MPs themselves were able of immune modulation due to the induction of the endogenous anti-bacterial factors capable of enhancing the neutrophil recruitment. In this study we tested several aspects of this hypothesis with the aim to better understand the mechanisms of anthrax immunopathology and to further characterize the safety and therapeutic potential of our MPs. In particular, our research revealed the role of MPs in overcoming the effect of anthrax infection on its major targets, pERK1/2 and DCs.

## Results

### Both the MPs and MP-loaded CKs contribute to the protection of mice against anthrax

**Figure 1:**
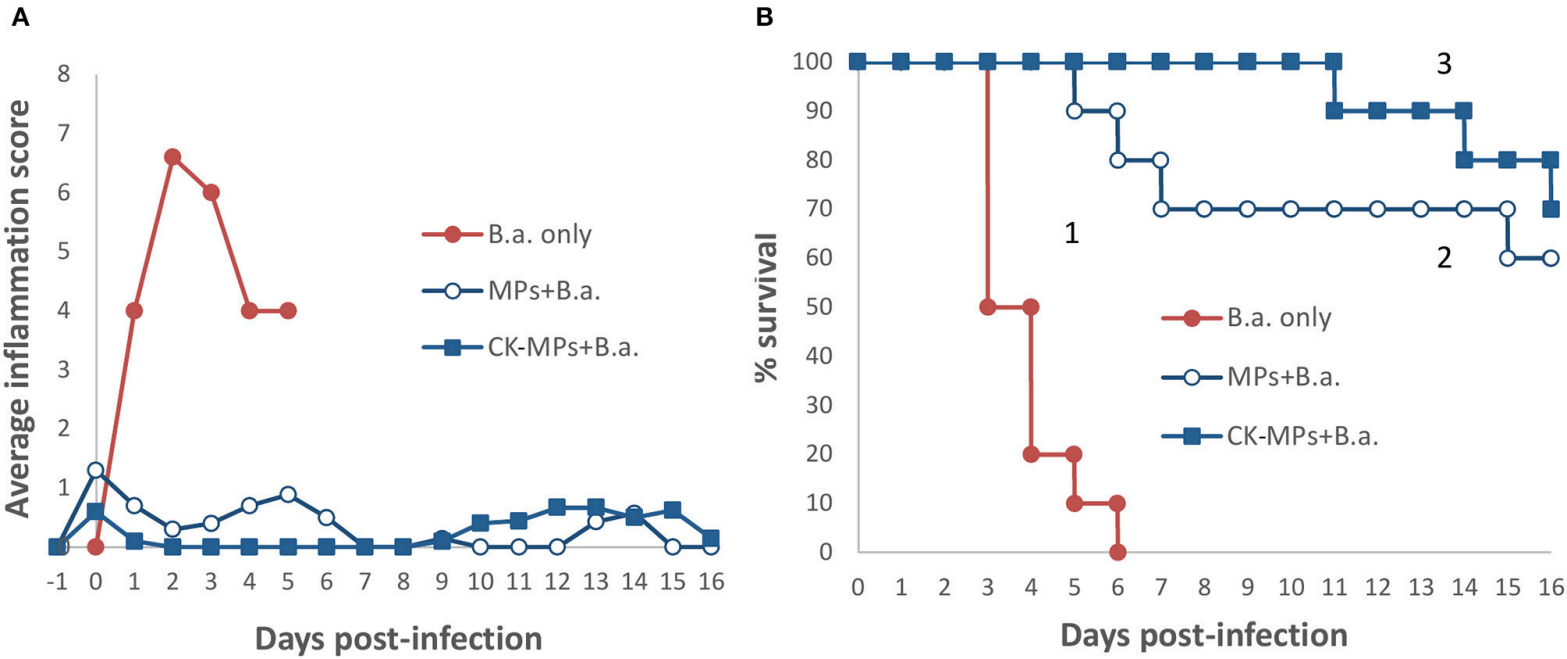
Pre-treatment of mice with CK-loaded MPs reduces footpad inflammation **(A)** and is protective against bacterial challenge with B.a. spores **(B)**. Mice were pre-treated with 50 μl of CK-MPs loaded with 1 μg/ml each of IL-8 and MIP-1α (squares, curve 3) or CK-MPs without loaded CKs (open circles, curve 2) injected into each hind footpad. Four hours later mice were challenged with 50 μl of B. a. spores (1.4 × 10^5^ spores per footpad) by the same route. Control mice were not pre-treated with CK-MPs (filled circles, curve 1). Average inflammation scores per mouse were assigned based on the thickness/redness of the hind footpads during the course of infection. Statistical significance of differences in survival using the Log Rank test was <0.0001 for curve 1 vs. 2, and 1 vs. 3.

We previously studied the biological properties of hydrogel MPs in mice subcutaneously challenged with a lethal dose of anthrax spores. It was found that the prophylactic administration of MPs before the spore challenge resulted in a remarkable reduction in mortality and associated inflammatory response. The anti-inflammatory effect appeared enhanced when the MPs were pre-loaded with the CKs (IL-8 and MIP-1α) known to attract immune cells of polymorphonuclear and monocyte origin (Singer and Sansonetti, 2004; Ramos et al., 2005). However, a high dose of spores (about 200 LD50) used prevented a reliable discrimination between the effects of loaded CKs and the MPs alone on mortality. In this study, the experiments at the reduced challenge dose (6.7 LD50) clearly demonstrated contribution of both components to survival (Figure 1). A single dose of the CK-MPs without any other intervention delayed the onset of mortality from 3 to 5 days and protected 60% of animals (*vs*. 0% without MPs). The MP-loaded CKs displayed even more prominent effect, compared to MPs alone, delaying the onset of mortality until day 10 while protecting 70% of spore-challenged animals. The MP pre-treatment almost completely eliminated a strong swelling and redness of the infected footpads observed in untreated animals.

### The MPs are not directly toxic to *Bacillus anthracis* spores, vegetative bacteria, or cultured raw 264.7 cells

Several hypotheses were considered by us to explain the effects of MPs and CK-MPs on the outcome of anthrax in our experiments. It was reported that nanoparticles can interact with and penetrate bacterial cells with unique bacteriostatic and bactericidal mechanisms (Seil and Webster, 2012). To test for the potential sporicidal, bactericidal, or bacteriostatic effect of CK-MPs, the latter were incubated with B.a. Sterne 34F2 spores in static cultures at 5% CO_2_ and 37°C, mimicking the conditions of spore germination and growth in the infected tissues *in vivo*. The impact of MPs on the amount of grown bacteria was determined based on bacterial metabolic activity using a fluorescent indicator Alamar Blue (resazurin). This method avoided problems associated with the fact that B.a. cells grow in long chains and therefore are difficult to quantitate using conventional microbiological procedures such as, plating onto solid agar and counting colonies. In the control cultures the spores were incubated in the absence of MPs. Since the MPs contain in their content a coupled triazine CB dye, additional control cultures included the dye at concentration corresponding to its content in the MPs. The active chlorine of the reactive dye was hydrolyzed during incubation in the 0.1 M carbonate buffer similar to its removal in the particle-coupling process. No effect of the MPs or the dye on the bacterial growth was found after the 24-h incubation (Figure S1A).

The administered MPs are likely to be partially engulfed by the phagocytic cells attracted to the site of infection (footpads, sentinel LNs). The RAW 264.7 macrophages are frequently used as the *in vitro* model to estimate a potential cytotoxic effect of MPs. We found that the cell viability measured as a capacity to reduce Alamar Blue dye was not influenced by the 24-h exposure to MPs with a coupled CB dye, as well as the soluble CB dye at concentration corresponding to its content in the MPs. However, higher CB concentrations showed partial cytotoxicity which occurred in a dose-dependent manner (Figure S1B).

### The MPs stimulate raw 264.7 macrophages to release immune response mediators

For the analyses of MP immune-stimulating potency the RAW 264.7 cells were exposed to MPs or control samples which included serum-free DMEM/F12, LPS from *Escherichia coli*, and the CB dye (preliminary hydrolyzed as described above). Supernatants were collected and analyzed using the Bio-Rad Bio-Plex Pro™ Mouse Cytokine 23-plex Assay for simultaneous determination of Eotaxin, G-CSF, GM-CSF, IFN-γ, IL-1α, IL-1β, IL-2, IL-3, IL-4, IL-5, IL-6, IL-9, IL-10, IL-12 p40, IL-12 p70, IL-13, IL-17A, KC, MCP-1, MIP-1α, MIP-1β, RANTES, and TNF-α.

Four cytokines (G-CSF, MCP-1, MIP-1α, and TNF-α) were found to be significantly up-regulated in the supernatants of MP-treated cells. The cytokines were detectable as early as 4 h and continued to increase in samples collected at 24 h (Table S1). The concentrations of IL-1α, IL-6, and KC calculated by the Bio-Plex analysis suggested upregulation of their expression, although all of the values were not statistically significant near the lower limit of detection (not shown). To confirm the Bio-Plex results, quantitative ELISAs were run for MIP-1α, MCP-1, TNF-α, and KC (Table 1). Robust responses were detected for MCP-1 and TNF-α. The release of MIP-1α was reliably increased as well, but was associated with a high level of background from unstimulated cells in control medium. KC demonstrated a low-level response to MPs, in agreement with previous reports on low capacity of RAW 264.7 cells to produce this chemokine in response to a particulate matter (Musah et al., 2012). *In vivo* upregulation of KC in the FP, however, was confirmed using immunohistochemical staining (data not shown).

**Table 1:** ELISAs of chemokine production by Raw 264.7 cells in response to CB-MPs.

	**4 h**		**24 h**	
**Cytokine**	**Control medium**	**CB-MPs**	**Control medium**	**CB-MPs**
MCP-1	69 ± 7	110 ± 53	220 ± 22	4, 040 ± 210
		77 ± 43		1, 650 ± 130
		58 ± 13		920 ± 30
MIP-1α	ND	ND	10, 920 ± 430	17, 870 ± 330
				10, 560 ± 210
				11, 940 ± 120
TNF-α	46 ± 6	1, 440 ± 100	59 ± 5	9, 880 ± 380
		1, 570 ± 120		10, 150 ± 160
		1, 980 ± 60		8, 800 ± 530
KC	ND	ND	<16	76 ± 20
			Below LL	76 ± 29
				26 ± 10

ELISAs were performed using supernatants of cells exposed to CB-MPs or hydrolyzed CB dye. The concentrations are in pg/ml ± CI (α = 0.05). The three values per cell in the CB MP columns represent MCP-1, MIP-1α, and TNF-α responses to 10, 5, and 2.5% MP bed volumes, notated from top to bottom. For KC, the three values represent responses to 10, 3.3, and 1% bead volumes. ND, analysis not done. LL, concentration below the lower limit of quantification.

To address the issue of the CB dye within the MPs influencing the magnitude and spectrum of cytokine stimulation, we examined the effect of soluble hydrolyzed CB dye. The dye was diluted to the concentration of allylamine used in the MP polymerization procedure to introduce primary amino groups into their content for CB coupling (40 μM in the 10% MP suspension). The Bio-Plex assay detected no significant influence of the dye at 4 h for any of the cytokines. Longer incubation (24 h) demonstrated higher stimulation; however, the cytokine levels remained much below that of MPs, indicating that the coupled dye was not a major contributor to the MP activity. Higher concentrations (10x) of dye significantly enhanced the levels of IL-12(p40), MIP-1α, MIP-1β, and TNF-α detected at 24 h in a dose-dependent manner (not shown). However, these results can only be used to demonstrate the potency of the free dye because such high concentrations (10x exceeding the stoichiometry of the allylamine groups) cannot be coupled to the MPs. Overall, the above data suggest a low stimulating activity of the coupled dye itself, taking into account that only a fraction of its concentration within the MPs is expected to be accessible to the cells. An additional experiment was conducted with allylamine MPs not coupled with the CB dye. As determined by ELISA, the KC-stimulating activity of the allylamine MPs (178 pg/ml after 24 h) was more than 2-fold higher than in the case of CB-MPs implicating surface chemistry as a strong contributor to the effect of MPs (Gupta et al., 2016).

### The MPs stimulate migration of neutrophils and DCs *in vivo*

**Figure 2:**
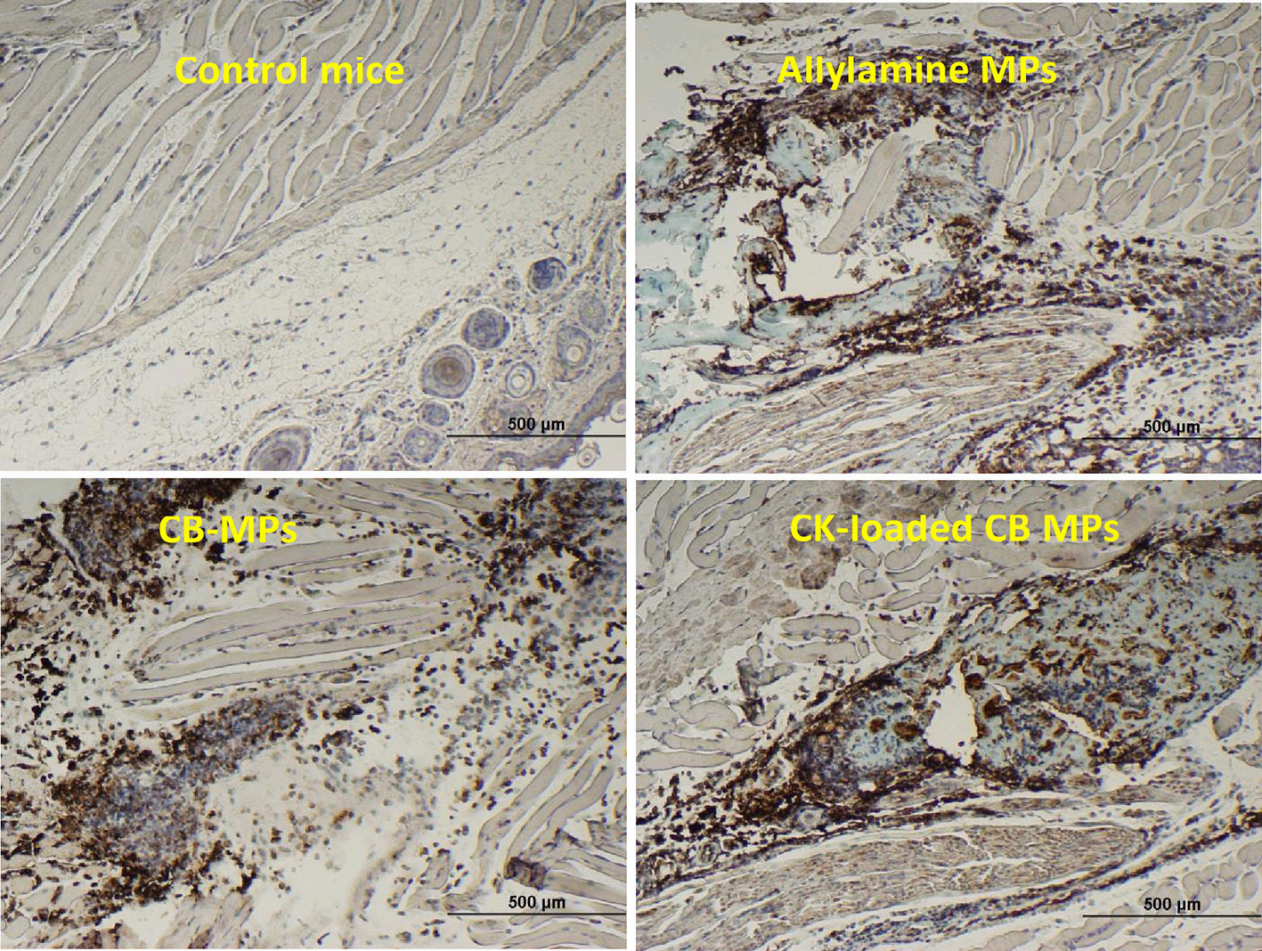
The MPs with or without coupled CB dye, as well as the CK-loaded CB-MPs induce migration of neutrophils to the subcutaneous tissue in MP-injected footpads. Mice were injected into hind footpads with 50 μl of indicated MP suspensions and euthanized after 24 h. The presence of neutrophil marker Ly6G was revealed immunohistochemically (as brown color of DAB stain using primary antibodies against Ly6G).

**Figure 3:**
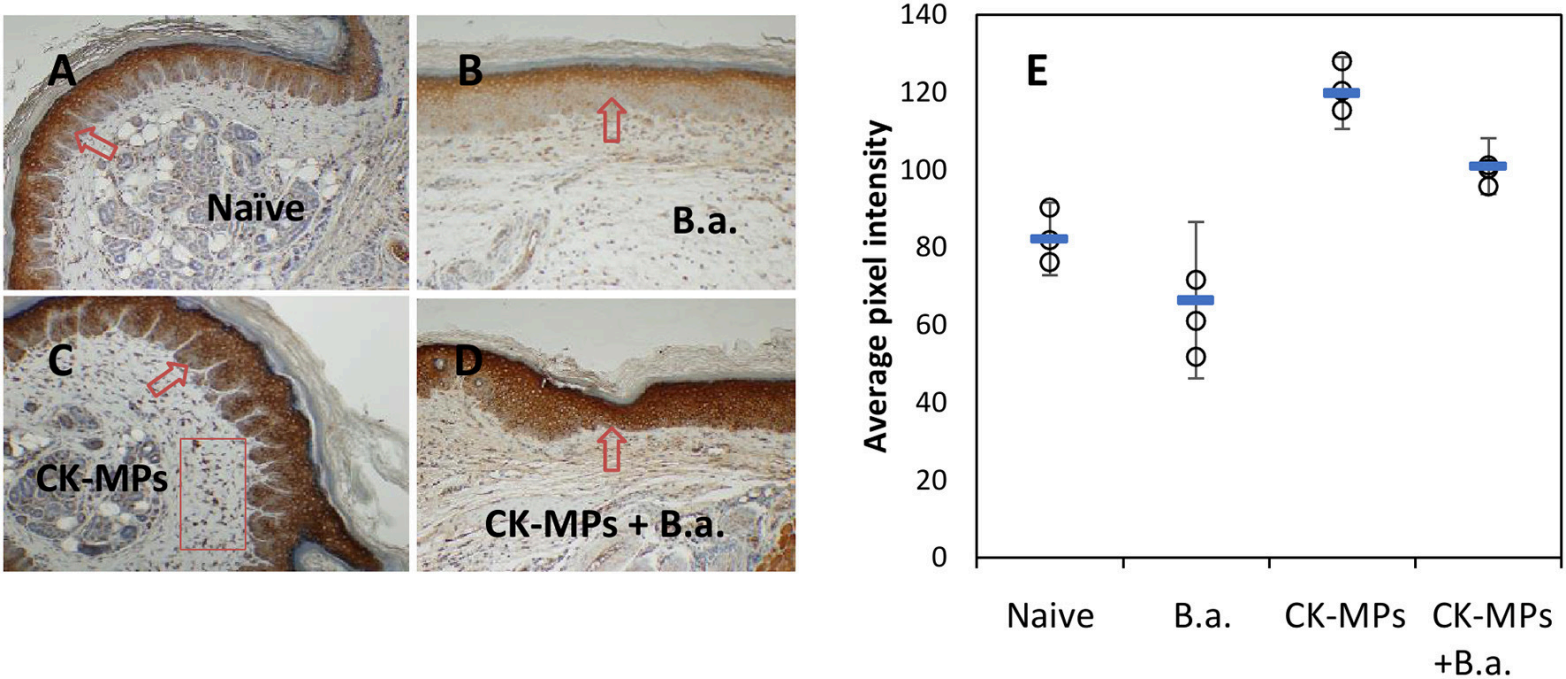
TNF-α in footpad skin of mice infected with B.a. **(B)** and pre-treated with MPs for 4 h before infection **(D)** in comparison with naïve mice **(A)**. Mice without B.a. challenge treated pre-treated with CK-MPs **(C)** were included. The levels of TNF-α in epidermis **(E)** were quantitated as described in the Materials and Methods. Mice were infected with 4 × 10^6^ spores per hind footpad and the immunohistochemical staining of TNF-α was performed at 24 h post infection. The arrows and rectangular region indicate the epidermal and dermal staining, respectively. Error bars are shown as mean (square marker) ± CI. Non-overlapping CIs indicate statistical reliability with α <0.05, *n* = 3.

Immunohistochemical staining of footpad slices using an antibody against neutrophil-specific antigen Ly6G showed that injection of MPs with the coupled CB dye (CB-MPs) or uncoupled allylamine MPs induced strong migration of neutrophils (Figure 2) similar to what was previously found in the case of MPs loaded with IL-8 and MIP-1α (Popova et al., 2016). We suggested that the appearance of neutrophils at the site of MP injection likely reflected the response of the skin cells (such as, vascular endothelial cells, keratinocytes, or fibroblasts) chemotactically drawing the inflammatory cells into the area (Mast and Schultz, 1996). In this scenario, the exogenous CKs loaded onto MPs might enhance the effects of endogenous neutrophil chemoattractants induced by MPs on the same or closely related pathways. Neutrophils arriving first to the site of injury or infection are known to be followed by macrophages. Both of these cell types serve as a major source of pro-inflammatory cytokines such as, TNF-α (Kanno et al., 2011). To assess participation of TNF-α in the effect of MPs *in vivo* suggested by the experiments with RAW 264.7 cells we carried out an immunohistochemical analysis of the cells expressing this cytokine. The cells stained positive for TNF-α in response to MPs were found in the regions of subcutaneous tissues broadly overlapping with the areas of neutrophil migration assessed by the marker Ly6G (Figure S2). These areas also contained cells positive for CD11b, CD11c, MHCII, and CD68, consistent with the presence of macrophages and DCs. Figure 3 shows the TNF-α was also readily detectable in the epidermal layer of naïve, MP-treated, and infected mice. The quantitation of images showed that the epidermal TNF-α level was downregulated in mice infected with B.a. (Figure 3B) in comparison with naïve mice (Figure 3A), although this effect did not reach statistical significance (Figure 3E). Pre-treatment with CK-MPs stimulated TNF-α expression in epidermis (Figures 3C, E) and prevented its downregulation by B.a. infection (Figures 3D, E).

The release of TNF-α in response to MPs *in vivo* and *in vitro* suggested that MPs could stimulate immune cell traffic. It was reported that neutrophil migration in immunized mice depends on the release of MIP-1α, which acts *via* the sequential release of TNF-α and LTB4 (Ramos et al., 2005). Neutralization of TNF-α in *Cryptococcus neoformans*-infected mice decreases the levels of both MCP-1 and MIP-1α (Huffnagle et al., 1996) and strongly ablates the migration of leukocytes (macrophages, neutrophils, and CD4+ T cells) demonstrating that TNF-α is a proximal mediator for chemokine induction. TNF-α is currently considered to be the key factor in the cascade activating skin epidermal Langerhans DCs (LCs; Epaulard et al., 2014). Macrophage- and neutrophil-derived TNF-α instructs DCs to prime immune responses which are associated with ERK1/2 pathway (Yanagawa et al., 2002; Epaulard et al., 2014).

**Figure 4:**
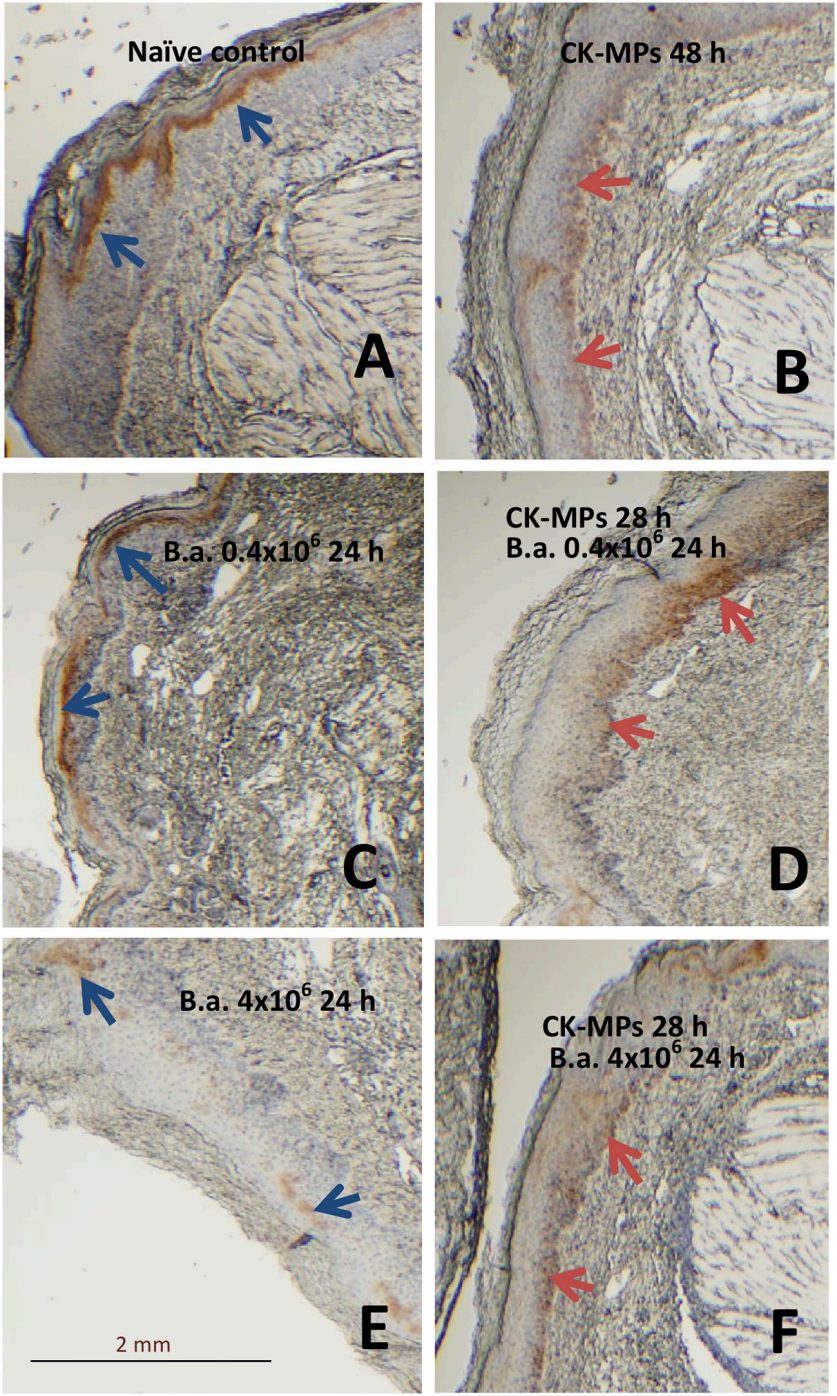
Transmigration of pERK1/2-positive cells through the epidermis of footpads (red arrows) in response to injected CK-MPs **(B,D,F)** with **(D,F)** or without **(B)** B.a. challenge. Mice were challenged with the indicated doses of B.a. spores at 4 h after CK-MP administration and immunohistochemical pERK1/2-specific antibody staining was performed at 24 h post infection. Controls included the naïve mice **(A)** and mice challenged with spores only **(C,E)**. The absence of cell migration is shown by blue arrows.

We found that the population of skin cells highly positive for the activated phosphorylated form of pERK1/2 demonstrated a behavior which could be explained by their migration through epidermis. These cells were readily detectable in the epidermis of naïve mice as a layer above stratum spinosum (Figure 4A). At 48 h post administration of the CK-MPs the pERK1/2-positive cells were noticed to localize closer to the border with dermis (Figure 4B). Similar migration took place in the B.a.-challenged mice after the 4-h pre-treatment with CK-MPs. The migration was accelerated and could be detectable as soon as 24 h post spore challenge (Figure 4D), while no migration took place at this time in infected mice without the MP pretreatment (Figure 4C). The infectious process resulted in the depletion of pERK1/2-positive cells increasing with the higher challenge dose (Figures 4C,E), and the MP pre-treatment showed a protective effect (Figures 4E, F).

**Figure 5:**
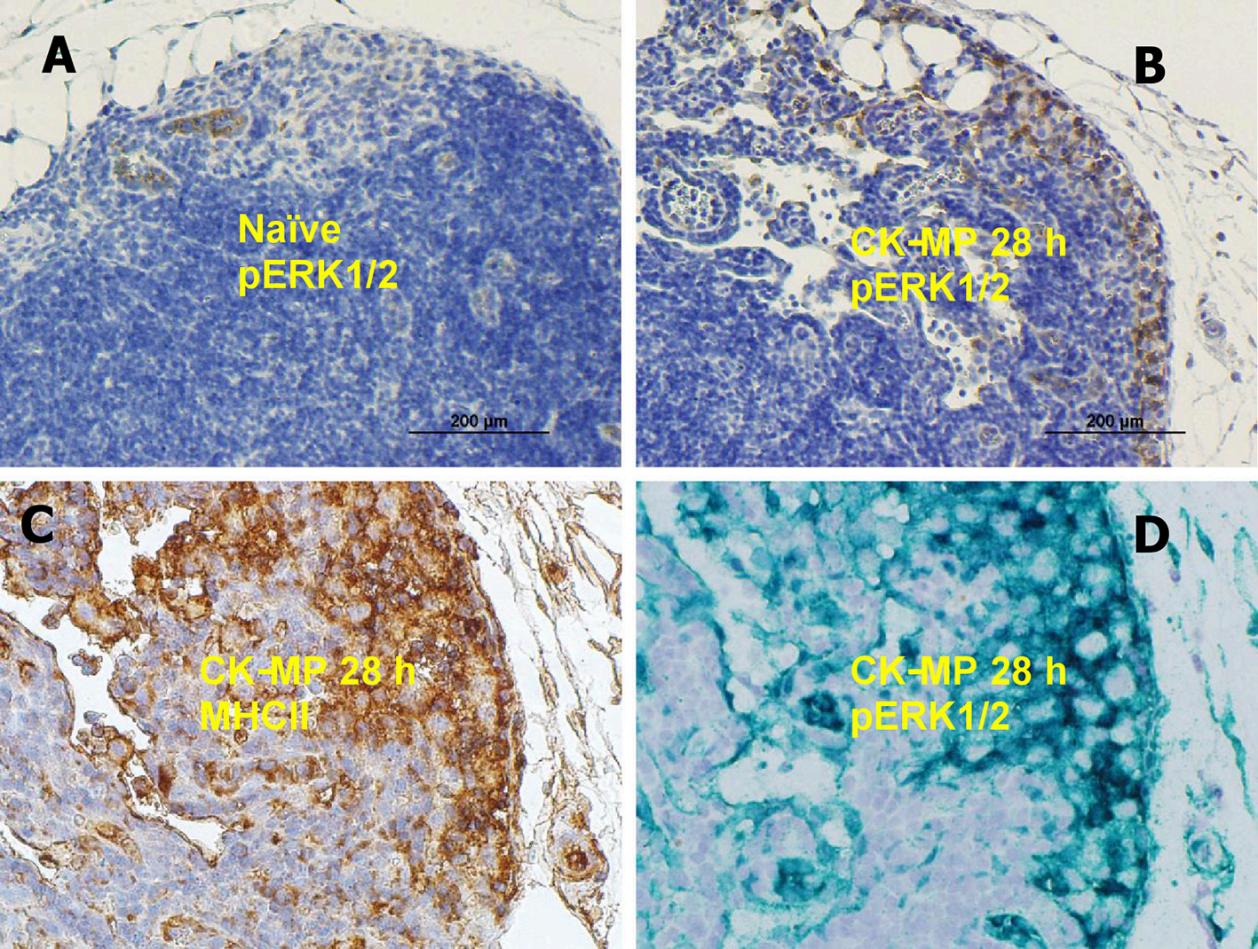
**(A,B)** Medullary region of popliteal LN in naïve mice **(A)** or after injection of CK-MPs for 28 h **(B)** 40x. Immunohistochemical staining with pERK1/2-specific antibody. **(A,B,D)** Overlapping patterns of immunohistochemical staining of LNs of mice injected into hind footpads with MPs for 28 h. Primary antibodies against MHC II **(C)** and pERK1/2 **(D)** were used to stain consecutive slides of LN tissue. The dyes were DAB **(A–C)** and Emerald Green **(D)**.

The DCs encountering antigens are known to migrate to regional LNs and display the MHC-class II molecules. Consistent with this, the number of pERK1/2-positive cells in the popliteal LNs increased after injection of MPs (Figures 5A,B). Patterns of immunohistochemical LN staining with antibodies against pERK1/2 and MHCII closely overlapped (Figures 5C,D). The pERK1/2-positive cells were identified as Langerhans DCs taken together their characteristic morphologic appearance in the skin (Figure S3), MHCII-positive phenotype (Figure 5), initial epidermal location, as well as the capacity to migrate through epidermis and LNs upon exposure to antigens.

## Materials and methods

### Materials

Unless otherwise indicated, all chemicals and reagents were from Sigma-Aldrich (St. Louis, MI, USA). The sandwich ELISA Ready-SET-Go! kits for mouse MCP-1 and TNF-α were from eBioscience. The MIP-1α ELISA kit was from RayBiotech. The KC ELISA kit was from R&D Systems. The carrier-free recombinant CKs from BioLegend (San Diego, CA, USA) were a mouse CCL3 (MIP-1α) and a human CXCL8 (IL-8). Endotoxin-free water was purchased from Life Technologies (Fredrick, MD, USA). *B. anthracis* Sterne strain 34F2 was from Colorado Serum Co.

### MP preparation

The MPs were synthesized and coupled to the CB dye as described (Popova et al., 2015). Briefly, poly(N-isopropylacrylamide) MPs containing co-polymerized allylamine were prepared via precipitation polymerization of N-isopropylacrylamide and N-N′-methylenebisacrylamide and allylamine in LPS-free water at 75°C for 3 h. After this time, the reaction was allowed to cool and the MPs were then pelleted by centrifugation. The centrifugation-dispersion process was repeated for a total of 5 times. The N4 Plus PCS Submicron Particle Analyzer (Beckman Coulter) was used to determine the particle size (500–600 nm) and the polydispersity index in water which was in the interval of 0.2–0.5. Cibacron Blue F3G-A (CB), the reactive triazine dye, was immobilized via direct reaction with the amine group of the allylamine units within the particles, displacing the chlorine on the di-substituted triazine ring of the dye in LPS-free 0.1 M bicarbonate buffer for 36 h at room temperature. After dye incorporation, the NPs were washed six times using tissue culture grade PBS diluted 1:3 with the endotoxin-free water. The absence of bacterial contaminants was demonstrated by plating 100 μl of final MP suspension onto Luria Broth agar plates and incubating them at 37°C for 48 h. A few drops of chloroform as a bactericidal agent were added to the final batch of NPs stored at 4°C. The endotoxin content of MPs (0.14 EU/ml) was measured with the Pierce Limulus Amoebocyte Lysate (LAL) Chromogenic Quantitation Kit (ThermoFisher) according to the manufacturer's protocols. The suspensions of CK-loaded NPs used for animal injections were prepared by incubating CB NPs (10% wet *v/v*) in PBS with a mixture of CXCL-8 and CCL3 (1 μg/ml each) at 4°C overnight. The suspensions were brought up to room temperature and injected into footpads of mice as described for animal challenge experiments below.

### Animal challenge experiments

All animal experiments were conducted under protocol #284 approved by George Mason University's Institutional Animal Care and Use Committee. In the experiments reported in Figure 1, three groups (*n* = 10 each) of female 6–8-week-old DBA/2 mice (Jackson Labs) received 50 μL intradermal injections of B.a. spores into each of the hind footpads. The B.a. dose per a footpad was 1.4 × 10^5^ spores. The Group 1 received no MPs, and the Groups 2 and 3 were pre-treated with 50 μl of MP suspension (10% of particle bed volume) 4 h before the spores by the same route as the spores. The MPs in the Group 2 contained no loaded CKs, and the MPs in the Group 3 were loaded with CKs. Control groups (*n* = 2–4) included mice injected with MPs only as described above. When indicated, the experiments were also carried out with 0.4 × 10^6^ or 4 × 10^6^ spores in 20 μl of PBS injected into each hind footpad. The animals were observed for 2 weeks. Semi-quantitative scores of footpad inflammation and edema were assigned as: 0 = no visible signs, 1 = initial signs of swelling and light redness in the footpad, 2 = prominent swelling and redness partially extending from the footpad to the ankle, 3 = strong swelling and redness extending to the whole ankle, 4 = extensive swelling and redness beyond the ankle. Log Rank and U-tests were used to estimate differences in mortality and inflammation between groups. It has to be noted that the delayed mortality of infected mice in the experiments with MPs may represent a new statistical event which changes the probability of dying during the cause of disease (such as, activation of the dormant spores) and therefore may complicate the interpretation Log Rank test (Dignam and Kocherginsky, 2008). No correction for potential competing events was made.

### Immunohistochemical analysis

For analysis of LNs and footpads the animals were injected with 20 μl of 1% Evans Blue dye in PBS into both hind footpads for visualization of LNs and euthanized with carbon dioxide 30 min later. Organs were placed in 10% neutral buffered formalin, embedded in paraffin blocks, sliced into 5 μm sections, and mounted onto glass slides for staining. Slides were subjected to antigen retrieval in sodium citrate buffer (pH 6) for 40 min at 95°C followed by incubation for 20 min at room temperature. All slides were stained with antibodies using a Dako autostainer and counter-stained with Mayer's hematoxylin (Sigma-Aldrich). To detect the presence of PMNs, tissue sections after antigen retrieval were incubated in 3% hydrogen peroxide in methanol for 5 min, the avidin and biotin blocking solutions from Dako each for 10 min, the protein blocking solution from Dako for 5 min, and the primary biotin-labeled anti-Ly6G antibody (Biolegend rat anti-mouse clone 1A8) at dilution 1:200 for 1 h. Following incubation with the primary antibody the Dako CSA kit reagents were used. Briefly, samples were incubated with a streptavidin-biotin-peroxidase complex binding to the primary biotinylated antibody via streptavidin. The bound peroxidase then catalyzed the precipitation of the biotinylated phenolic compound onto the sample for amplification of the number of biotin molecules available for binding with the streptavidin-peroxidase added next. Antibody staining was completed with a 5-min incubation with 3,3′-diaminobenzidine tetrahydrochloride (DAB) and followed with counter-staining using Mayer's hematoxylin. The non-biotinylated antibodies against TNF-α (Abcam ab9739), MHCII (Invitrogen PA5-22113), MCP1 (Abcam ab25124), pERK1/2 (Cell Signaling 9101), CD68 (Abcam ab125212) were used. Antigen-retrieved sections were incubated with 3% hydrogen peroxide in methanol for 5 min, blocked with Dako protein block for 5 min, and incubated with the antibody diluted 1:200 for 1 h (4 h for TNF-α) or 1:100 for 2 h (CD68), followed by Dako anti-rabbit EnVision + HRP-Labeled Polymer (Dako). As before, antibody detection was finalized by incubation with DAB or Emerald Green and counter-staining with hematoxylin. For a quantitation of the DAB stain intensity the images were separated into the RGB colors and the inverted green color layer was used to identify the stained areas on each image using ImageJ software from the National Institutes of Health. A total of 16 independent images entirely covering the stained areas in each tissue section from two to four mice were processed per a particular experimental condition. The average pixel density was calculated.

### Effect of the MPs on B.a. spores and vegetative bacteria

The MPs or the coupled CB dye contained in the particles were tested for their effect on the B.a. spore germination and vegetative bacterial growth. The MPs or CB dye were incubated in a 2-ml total volume with B.a. Sterne 34F2 spores per well in a 12-well plate. Wells without spores were included as controls for each sample. The final concentrations in the appropriate wells were: 8 × 10^2^ spores/ml, 5% bed volume of MPs, or the CB dye diluted to concentration corresponding to its expected content in the MPs after coupling (concentration of allylamine groups). The reactive chlorine of the dye was hydrolyzed during its incubation in a carbonate buffer to account for its removal in the coupling reaction. MPs or dye were incubated with spores for 24 h or for 0 h (spores added to medium immediately before collecting samples) at 37°C. After incubation, 1 ml of each sample was collected and pelleted at 8,600 g for 10 min. Supernatants were removed and replaced with 1 ml of Alamar Blue in serum-free medium. Samples were incubated at 37°C with Alamar Blue for 1 h, centrifuged again, and 200 μl from each supernatant were plated 3x in a 96-well plate. Fluorescence of the dye was read at 530/590 nm using a plate fluorimeter. Fluorescence measurements were normalized to control groups containing bacteria only to represent viability.

### Effect of the MPs on raw 264.7 cell viability

To test the effect of MPs on RAW 264.7 cell viability the cells were grown to 60–70% confluency in a 96-well plate in DMEM/F12 containing 10% fetal calf serum. Cells were starved for 1 h in 100 μl/well of serum-free DMEM/F12. Medium was then removed and replaced with 100 μl samples. Samples included control serum-free medium, MPs diluted to give 10, 3.3, or 1.1% of bed volume, and the hydrolyzed CB dye diluted to give 10-fold, 3.3-fold, or 1.1-fold molar excess over its concentration in the MPs. The MPs were pre-washed three times in PBS, one time in serum-free DMEM/F12, and then resuspended in fresh serum-free DMEM/F12. To account for a possible effect of LPS contamination, LPS from *E. coli* L3012 serotype O111:B4 was used at 10 μg/ml. After cell exposure for 24 h the samples were removed, cells were washed three times with 100 μl of warm PBS, and 200 μl of Alamar Blue in CSFM were added into each well. Fluorescence was measured after 2 h at 530/590 nm. Fluorescence measurements were normalized to control groups containing RAW 264.7 cells only to represent viability.

### Effect of the MPs on the release of immune mediators by raw 264.7 cells

RAW 264.7 cells were grown as 2-ml cultures per well in 12-well plates until 80–90% confluency using DMEM/F12 medium supplemented with 10% fetal bovine serum. Cells were serum starved in 1 ml serum-free DMEM/F12 for 1 h and then exposed to 1 ml of samples which included the MPs (at 10% pellet volume) with or without a CB dye coupled with free allylamine amino groups of MPs. Controls included serum-free DMEM/F12, 10 μg/ml LPS from *E. coli* L3129 serotype O127:B8, and the hydrolyzed CB dye diluted to concentrations corresponding to about 10-fold, 3-fold, and 1-fold molar excess over its content in MPs. At certain times, supernatants were collected and centrifuged to pellet the MPs. All supernatant samples were supplemented with 0.5% BSA for protein stabilization, frozen at −20°C, and then analyzed using ELISA or the Bio-Plex Pro™ Mouse Cytokine 23-plex Assay (Bio-Rad) according to the manufacturer's protocol. The kit's standards were reconstituted and diluted in serum-free DMEM/F12 containing 0.5% BSA. Samples and standards were loaded onto the kit's magnetic beads for 30 min, washed, and incubated with the secondary detection antibody for 30 min, followed by streptavidin-peroxidase conjugate for 10 min. After washing and final re-suspension in the kit's assay buffer, the magnetic beads were analyzed using the Bio-Plex machine at low photomultiplier settings (RP1). For ELISA, supernatants for detection of MCP-1 after 4-h and 24-h cell exposure to the MPs were diluted from 5- and 10-fold, respectively, with serum-free DMEM/F12 with 0.5% BSA. Samples for detection of TNF-α were diluted 10- and 50-fold, respectively. Only the 24-h supernatants were analyzed for MIP-1α and KC. The supernatants for MIP-1α analysis were diluted 5-fold and undiluted for KC analysis. The supernatants of controls (cells only) were undiluted for detection of TNF-α, MCP-1, and KC, whereas those for MIP-1α analysis were diluted 5-fold.

## Discussion

MPs and nanoparticles of different types attract considerable attention as vehicles for drug delivery or nanoantibiotics displaying direct antibacterial, antifungal, and antiviral activities. Another avenue of active research is the interaction of MPs with the immune system exploring the modulation of host response as therapeutic approach in a broad range of diseases, adverse health conditions, or in the course of vaccination. We recently demonstrated the protective effect against anthrax infection in mice pre-treated intradermally with CK-MPs and then challenged with B.a. Sterne spores. In this study using optimal spore doses (reduced in comparison with previous experiments) it was possible to demonstrate that the time of disease onset and the increased survival of the MP-pretreated mice were dependent on both the activity of MPs *per se* and the CKs loaded onto the MPs. In the case of CK-MPs the mortality was delayed by additional 8 days. The survival was associated with a strong suppression of the inflammatory reaction in the spore-injected footpads. It is remarkable that such a profound impact on the course of disease resulted from a single dose of CK-MPs without any additional therapeutic intervention. Even seemingly inert MPs without a coupled dye caused a reliable increase in survival and a decrease in inflammation. The origin of mortality beyond day 10 in the CK-MP-treated group requires further analysis; it may represent an activation of dormant spores after the innate protective effect of MPs waned, but the induced immunity was insufficient to eliminate them entirely.

The observed effects could not be explained by the direct antimicrobial activity of MPs as it was previously demonstrated in the course of *Staphylococcus aureus* treatment with zinc oxide nanoparticles (Seil and Webster, 2012). We also didn't detect any substantial direct cytotoxicity of the MPs or the CB dye in relevant concentration toward cultured RAW 264.7 cells frequently used for evaluation of nanoparticle materials, alleviating concerns about adverse effects of our MPs in potential medical and scientific applications. On the other hand, multiplex analysis of 23 cytokines confirmed by specific ELISAs revealed upregulation of G-CSF, MCP-1, MIP-1α, and TNF-α released by RAW 264.7 cell in response to MPs. These data led us to suggest that the effect of MP administration to mice resulted from the immune-modulating capacity of the MPs rather than their direct antibacterial action. The absence of IL-1β response indicated that neither the MP scaffold nor the coupled dye activated the inflammasome in RAW 264.7 cells, in contrast with what was observed in the case of several nanomaterials (Palomäki et al., 2010; Santos et al., 2013), including the MPs loaded with LPS (Meraz et al., 2012; Li and Boraschi, 2016). It also showed that the LPS commonly contaminating the MP preparations (Li and Boraschi, 2016) was unlikely to contribute to our results.

Our observations with RAW 264.7 macrophage-like cells parallel several reported examples of the immune response to MPs by macrophages and DCs. The activation of innate responses in macrophages by poly(lactide-co-glycolic acid) nanoparticles improves the outcome of infection with *Leishmania braziliensis*. The decreased parasite load *in vitro* is associated with the augmented production of nitric oxide, superoxide, and IL-6. An increased release of TNF-α, CCL2/MCP-1, and CXCL1/KC also takes place, resulting in macrophage and neutrophil recruitment *in vitro* (Santos et al., 2013). Similar to macrophages, strong stimulated production of CKs and inflammatory cytokines, as well as up-regulation of co-stimulatory molecules, are also reported in the case of MPs, which are efficiently internalized by DCs (Broos et al., 2010). The 200 nm-sized biodegradable poly(gamma-glutamic acid) MPs are activators of human monocyte-derived DCs producing IL-8, MIP-1α, MIP-1β, and MCP-1. In addition, TNF-α and IL-1β are detected, albeit at a lower level.

The carboxylated polystyrene MPs were reported to modulate DC homeostasis, thereby promoting a persistent enhanced state of immune readiness to a subsequent infectious challenge (Xiang et al., 2015). Intradermal administration of these MPs induces anti-inflammatory cytokines, CKs and growth factors, increases numbers and proportions of DCs in the draining lymph nodes (LNs), and increases the capacity of bone marrow to generate DCs. Consistent with this observation, mice pre-injected with the MP show enhanced ability to generate anti-malarial immunity.

Neutrophil recruitment in immunized mice is shown to depend on MIP-2 inducing the sequential release of MIP-1α, TNF-α, and LTB4 (Ramos et al., 2005, 2006). MCP-1 and MIP-1α mediate firm adherence and subsequent transmigration of neutrophils *via* protein synthesis and secondary generation of leukotrienes and the platelet-activating factor, which in turn directly activate neutrophils (Reichel et al., 2009). Consequently, the activated neutrophils (as well as macrophages) recruited to the tissues can release TNF-α which instructs skin LCs to initiate immune responses (Epaulard et al., 2014). Taken together, the above features of the MP interaction with the immune system resulting in the release of CKs and recruitment of immune cells represent a likely explanation of our observations on the protective effect of MPs. Our data *in vivo* confirmed recruitment of neutrophils (Figure 2), upregulation of TNF-α level in the epidermis (Figure 3), and migration of epidermal cells with characteristic features of skin DCs (LCs) through epidermis in response to MPs (Figures 4, 5). The subcutaneous areas of MP-injected tissues stained positive for macrophages and DCs in the areas of neutrophil recruitment (Figure S1).

Secretion by neutrophils of the MIP-1α resulting in DC recruitment in the case of *Leishmania major* is shown to confer protection against intradermal inoculation of the parasite, as a markedly decreased DC recruitment is observed in mice depleted of neutrophils or deprived of the capacity to produce MIP-1α (Charmoy et al., 2010). The release of MIP-1α and IL-8 may be secondary to the induction of initial migration of DCs and monocytes as it was shown for acute-phase protein amyloid A produced during infection by hepatocytes, adipose tissue, endothelial cells, and macrophages. These CKs enhance DC migration and promote the recruitment of distant cells (Gouwy et al., 2015). From this standpoint, the exogenous MIP-1α and IL-8 loaded onto MPs are expected to enhance the effects of the endogenously induced ones, in agreement with the additional protection conferred by these CKs in B.a. challenge experiments (Figure 1).

We previously showed that our MPs are actively trafficked to the regional LNs from the injected footpads. Here, we found that the MPs increased pERK1/2 expression in the LNs by cells positive for the MHC class-II marker indicating their DC phenotype. The migration was not detected without MPs and was associated with the increased levels of TNF-α known to be required for the LNs trafficking. The infectious process resulted in the elimination of ERK1/2 activation and the reduction in the TNF-α epidermal staining, in line with the well-known capacity of anthrax toxins to target DCs and downregulate the MAPK signaling (Guichard et al., 2012), while administration of MPs had a protective effect.

In conclusion, our findings show for the first time that the poly(N-isopropylacrylamide) open-mesh MPs containing co-polymerized allylamine or covalently-bound CB dye behave as potent immune stimulators. The dye-coupled MPs were capable of altering the course of infectious disease, and their potency was enhanced by loaded CKs. Detailed examination of the specific roles the immune cell types and their subpopulations, including the DCs, play in the effect of MPs is forthcoming. It will be intriguing to evaluate the effect of MPs with different coupled (surface or external) chemistries against pathogenic bacteria other than B.a. used under different modes of MP administration.

## Author contributions

Contributed to conception and design; SP, LL, and VE. Contributed to acquisition, analysis, and interpretation of data: AT, TP, and SP. Drafted and/or revised the article: SP, AT, and TP.

### Conflict of interest statement

The authors declare that the research was conducted in the absence of any commercial or financial relationships that could be construed as a potential conflict of interest.
