# Supplementary material for: Immune-modulating Activity of Hydrogel Microparticles Contributes to the Host Defense in a Murine Model of Cutaneous Anthrax
Source: Front Mol Biosci. 2017 Aug 28;4:62. doi: 10.3389/fmolb.2017.00062 (PMC5581330; doi:10.3389/fmolb.2017.00062)
Supplement: Supplementary file 1 [file Image1.PDF]

A

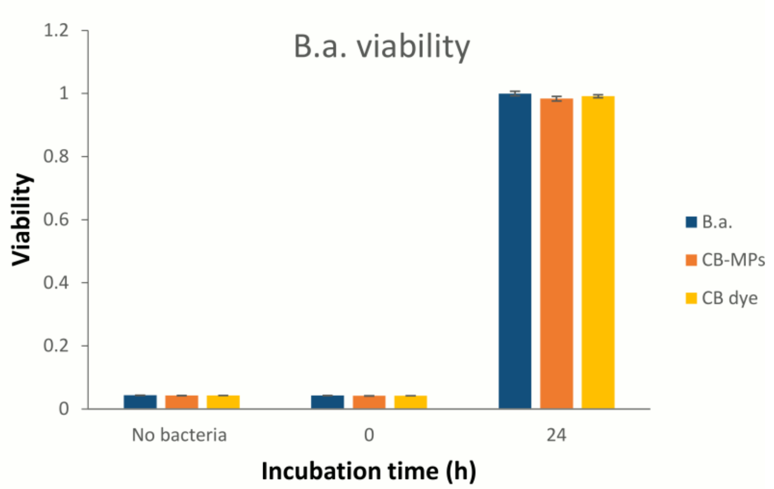

B

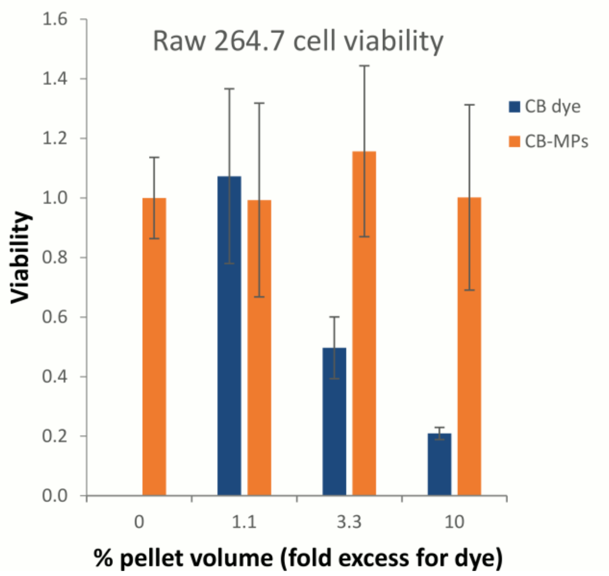

Figure S1. Effect of CB-MPs and hydrolyzed CB dye on viability of B.a. vegetative bacteria (A) or Raw 264.7 cells (B) after 24-h incubation at 37°C, 5% CO_2. (A) Static cultures were seeded with B.a. spores and bacterial growth was detected by Alamar Blue fluorescence after incubation with 5% (bed volume) of CB-MPs. (B) Raw 264.7 cells were incubated with the indicated amount of CB-MP and cell viability was detected by Alamar Blue similar to (A). The CB dye was used at concentration corresponding to its expected content in the MPs after coupling (A) or at the indicated fold excess over the amount of coupled dye (B). Error bars indicate mean±CI (α=0.05).
