# Supplementary material for: Immune-modulating Activity of Hydrogel Microparticles Contributes to the Host Defense in a Murine Model of Cutaneous Anthrax
Source: Front Mol Biosci. 2017 Aug 28;4:62. doi: 10.3389/fmolb.2017.00062 (PMC5581330; doi:10.3389/fmolb.2017.00062)
Supplement: Supplementary file 2 [file Image2.PDF]

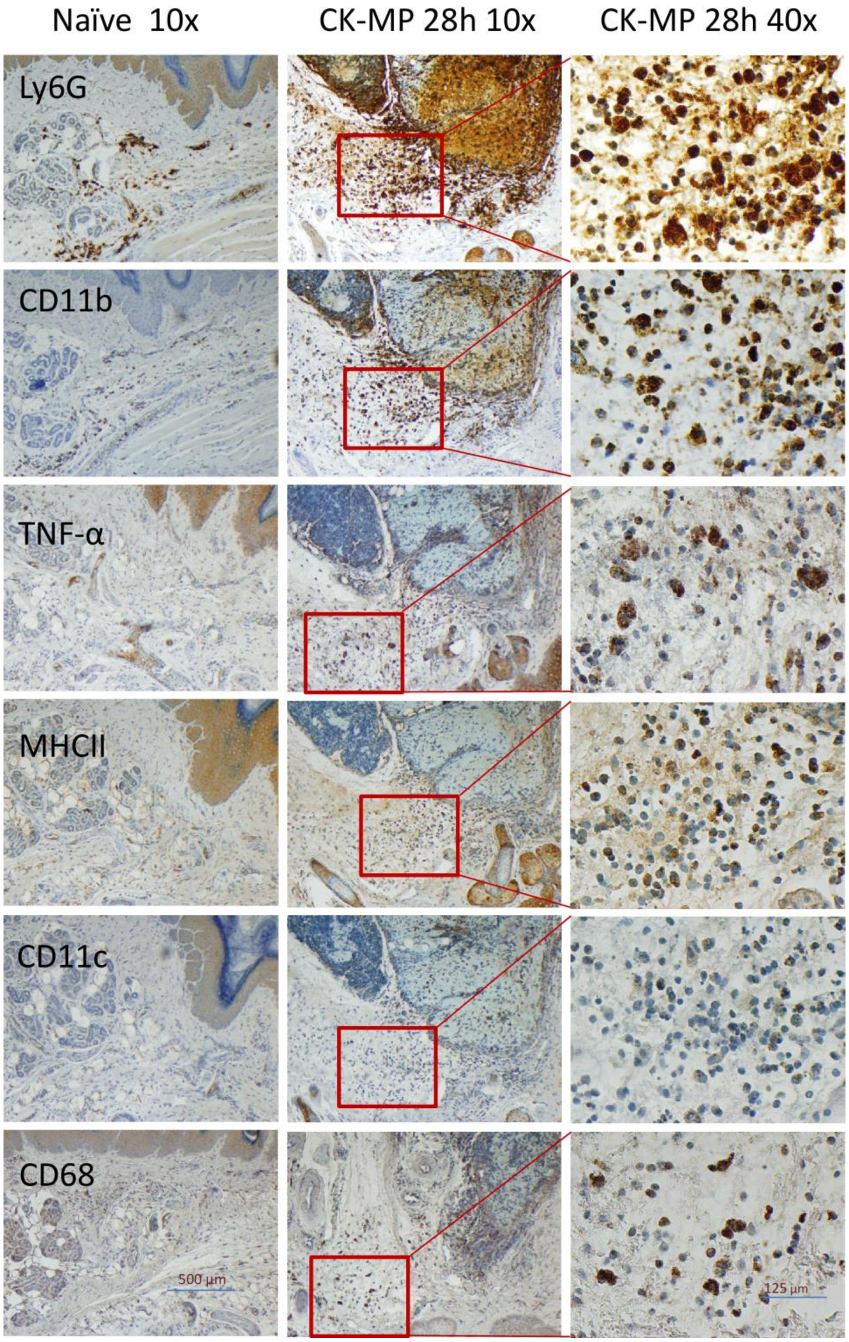

Figure S2. Neutrophil infiltration into footpads in response to intradermal administration of CK-MPs is associated with the increased levels of immune cells immunochemically stained positive (brown color of DAB stain) for Ly6G, CD11b, TNF-α, MHCII, CD11c, and CD68. Consecutive tissue slices from representative naïve and CK-MP-treated mice were analyzed at 28 h post injection. The rectangular regions in the middle column are shown at higher magnification in the right column.
