# Supplementary material for: Immune-modulating Activity of Hydrogel Microparticles Contributes to the Host Defense in a Murine Model of Cutaneous Anthrax
Source: Front Mol Biosci. 2017 Aug 28;4:62. doi: 10.3389/fmolb.2017.00062 (PMC5581330; doi:10.3389/fmolb.2017.00062)
Supplement: Supplementary file 3 [file Image3.PDF]

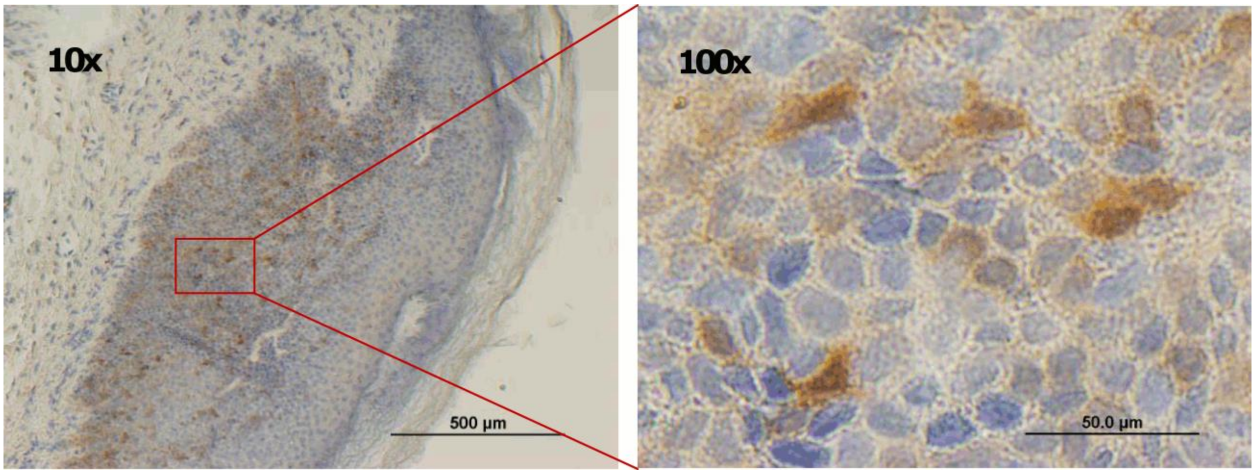

Figure S3. Immunohistochemical staining with antibody against pERK1/2 in epidermal section of footpads in naïve mice displaying Langerhans phenotype of pERK1/2-positive cells identified by a brown color of DAB stain. The rectangular region of the image in the left panel is shown at higher magnification in the right panel.
