# Supplementary material for: Immune-modulating Activity of Hydrogel Microparticles Contributes to the Host Defense in a Murine Model of Cutaneous Anthrax
Source: Front Mol Biosci. 2017 Aug 28;4:62. doi: 10.3389/fmolb.2017.00062 (PMC5581330; doi:10.3389/fmolb.2017.00062)
Supplement: Supplementary file 4 [file Table1.DOCX]

**Table S1**. Bio-Plex Pro™ 23-plex Assay of cytokine production by Raw 264.7 cells

|  | **CB MPs (10%)** | | | | **CB dye** | |
| --- | --- | --- | --- | --- | --- | --- |
|  | **4 h** | | **24 h** | | **4 h** | **24 h** |
| **Cytokine** | **Control**  **medium** | **MPs** | **Control**  **medium** | **MPs** | **1x**  **concentration** | **1x**  **concentration** |
| **G-CSF** | <2 | 53±2 | 53±5 | 14,840±30 | 26±3 | 181±1 |
| **MCP-1** | 1594±1 | 5120±10^*^ | 1,920±10 | 13,230±20^*^ | 1,314±2 | 2,598±1 |
| **MIP-1α** | 22,340±380 | 31,760±360^*^ | 80,080±170 | >137,000 | 26,108±4 | 84,800±400 |
| **TNF-α** | 90±1 | 1,780±5 | 101±1 | 34,510±40 | 77±0 | 158±1 |

Cells in serum-free DMEM/F12 were exposed to CB MPs (at 10% or 2.5% bead volume) or hydrolyzed CB dye (at concentration equal to that in MPs) for 4 h or 24 h. Only cytokines which were significantly increased in at least one of the MP- or dye-treated samples relative to the control are shown. All values represent the cytokine concentrations (pg/ml ± SD) in samples which were tested diluted 10-fold in serum-free medium. ^*^ indicates the response to MPs at 2.5% bed volume.
